# Digitizing ESP beyond chalk and talk in the English for tourism classroom

**DOI:** 10.1038/s41598-026-55864-4

**Published:** 2026-06-08

**Authors:** Ali Ahmad Al-Barakat, Rommel Mahmoud AlAli, Ruba Fahmi Bataineh, Sarah Bader Mohsen Alotaibi, Nada Ibrahim Alribdi, Ali Khalifa A. Abdullatif, Rula Fahmi Bataineh

**Affiliations:** 1 Department of Education, University of Sharjah, 27272 Sharjah, United Arab Emirates; 2 Faculty of Educational Sciences, Yarmouk University, Irbid, 21163 Jordan; 3 The National Research Center for Giftedness and Creativity, King Faisal University, 31982 Al-Ahsa, Saudi Arabia; 4 College of Education and Human Development, Princess Nourah bint Abdulrahman University, Riyadh, 11671 Saudi Arabia; 5 Department of Arabic Language, College of Arts, King Faisal University, Al Ahsa, Saudi Arabia; 6 Jordan University of Science and Technology, Irbid, 21163 Jordan

**Keywords:** Digital technologies, English for tourism, ESP, Tour guiding, Tourism education, Virtual learning environments, Psychology, Human behaviour

## Abstract

This study explores faculty members’ perceptions in tourism education programs regarding the use of digital technologies to enhance English language proficiency among learners preparing for careers as tourist guides. The study sample consisted of sixty-five instructors from tourist guide training institutions. Data were collected through semi-structured interviews. The research instrument was developed with reference to English for Specific Purposes (ESP) frameworks to ensure alignment with the linguistic and professional demands of the tourism sector. Data were analyzed using thematic analysis to identify prevailing pedagogical approaches and patterns of technological integration. The findings reveal that faculty members recognized the value of incorporating advanced digital tools, such as simulations, virtual tours, digital assessments, and interactive platforms, into language instruction. These tools were perceived by participants to support learners’ fluency, context-specific vocabulary, self-confidence, and motivation. Moreover, participants perceived the shift to digital methodologies as improving instructional relevance and better aligning educational experiences with industry expectations, potentially increasing students’ preparedness for real-world interactions. The study concludes by emphasizing the need for targeted professional development to equip instructors with the digital competencies required for effective English instruction in tourism contexts.

## Introduction

Tourism is widely recognized as one of the fastest-growing global industries, with significant contributions to national economies through job creation in sectors such as transportation, hospitality, and ^1–3^. Notably, tourism also supports development in marginalized and rural areas by stimulating commerce and preserving cultural heritage through traditional crafts and creative enterprises^4–6^. Beyond its economic role, tourism plays a key role in promoting intercultural understanding by fostering positive exchanges and reducing stereotypes among diverse populations^7,8^.

As global tourism continues to expand, destinations are increasingly receiving visitors from diverse linguistic and cultural backgrounds. Effective communication has become essential, not only to meet the expectations of international guests but also to build meaningful interpersonal connections rooted in mutual respect and cultural awareness^9–11^. English, as the dominant global lingua franca, facilitates many of these interactions, especially since recent advancements in language technologies have broadened communication beyond speech, with digital translation tools enabling rapid and accessible cross-cultural exchanges^11,12^.

Tour guides, as front-line representatives of their countries, play a pivotal role in shaping tourists’ perceptions. They serve not only as communicators of information but also as cultural ambassadors and socio-cultural mediators^1,12^, which requires strong English language skills and high levels of interpersonal intelligence. However, many non-native guides struggle with pronunciation, vocabulary, and listening comprehension, especially when interacting with tourists who speak English with varied accents^13–16^. These communication gaps may compromise service quality, reduce tourist satisfaction, and potentially damage the reputation of the destination^17–20^.

The root of this problem lies partly in outdated pedagogical approaches which often fall short of addressing the practical communication needs of the field. Traditional English for tourism instruction tends to isolate grammar and vocabulary from authentic usage, treating language as a fixed system rather than a dynamic communicative tool^7,21–23^. This disconnect between classroom learning and real-world application often results in graduates who have the theoretical knowledge but are inadequately equipped to meet the communicative demands of the tourism sector^7,22^. Essential communicative competencies, such as storytelling, negotiation, public speaking, and active listening, are frequently neglected despite being crucial for guiding tourists in spontaneous, emotionally resonant interactions^24,25^.

In contrast, digital technologies now offer a promising solution. Immersive tools, including virtual and augmented reality, role-play applications, and online professional English courses, provide practice-oriented environments that closely simulate real-life tourism scenarios^26,27^. These technologies expose learners to diverse accents, enhance pronunciation through repeated practice, enable self-correction and immediate feedback, reduce performance anxiety, and foster greater fluency in spontaneous interactions^28,29^.

Research indicates that mobile applications significantly enhance English for tourism instruction by preparing learners for authentic communication within tourism contexts through contextualized and experience-based learning scenarios^2,30–34^. Rather than serving merely as supplementary tools, digital technologies represent a pedagogical shift toward experiential learning by integrating simulation, real-world immersion, and real-time feedback. Such developments demonstrate that effective English communication contributes directly to tourist satisfaction, repeat visitation, and destination loyalty^35,36^.

However, communicative competence extends beyond linguistic proficiency to include intercultural literacy. Previous studies (e.g., ^10,37^) emphasize the importance of training tourism professionals to interpret culturally embedded symbols, understand social conventions, and engage respectfully across cultural boundaries. Speaking, in particular, has been identified as the most essential of the four foundational language skills for tourism professionals. Nonetheless, persistent challenges, including limited intercultural awareness, insufficient vocabulary, inappropriate language use, and difficulty understanding foreign accents, often lead to misunderstandings and communication breakdowns (e.g., ^7,37^).

To bridge the gap between language instruction and the practical demands of the tourism industry, scholars (e.g., ^38,39^) advocate for applied and learner-centered pedagogical approaches. Project-based learning, for instance, has proven effective in promoting creativity, critical thinking, and the practical application of knowledge, particularly in tourism and hospitality education^40,41^. Such approaches not only strengthen learners’ professional preparedness but also create authentic opportunities for meaningful language use, thereby narrowing the divide between classroom learning and workplace communication.

Unlike previous ESP and digital learning studies that primarily focus on technology adoption or general language outcomes, the present study contributes new knowledge by examining how digitally enhanced, tourism-oriented ESP instruction can bridge the gap between classroom-based language learning and the communicative realities of the tourism workplace. The study further emphasizes the integration of intercultural competence, authentic tourism simulations, and hybrid pedagogical models as interconnected dimensions essential for preparing vocational tourism learners for real-world professional interaction. In doing so, it offers a more comprehensive perspective on digital ESP instruction in tourism education, particularly within vocational learning contexts where communicative confidence, cultural responsiveness, and employability skills are equally critical.

Despite these pedagogical advancements, many vocational learners continue to struggle with low confidence and limited vocabulary, factors that hinder their ability to use advanced language forms effectively. Such deficiencies often result in reduced self-esteem and restricted communicative development. To address these challenges, early and continuous instruction in oral and written communication remains essential (e.g.,^42^), as strong language proficiency constitutes a fundamental prerequisite for long-term career advancement in the tourism sector.

At the same time, the integration of digital learning presents both opportunities and challenges. Although digital platforms provide flexibility, learner autonomy, and access to diverse educational resources, barriers such as inadequate infrastructure, limited digital literacy, and reduced interpersonal interaction (e.g., ^43–46^) may significantly constrain their effectiveness, particularly in a field such as tourism, where emotional intelligence and face-to-face communication remain central.

Despite these challenges, the role of the educator remains pivotal. Effective instruction depends on educators’ ability to integrate traditional pedagogical approaches with emerging technologies in ways that are both pedagogically sound and technologically responsive (e.g., ^47^). Faculty members must therefore lead the transition toward innovation while preserving the interpersonal and cultural dimensions that are fundamental to the preparation of competent tourism professionals.

### Problem and Significance of the study

Proficiency in English is fundamental to most professional disciplines, but in tourism, it is particularly crucial due to the need to communicate with guests from diverse linguistic and cultural backgrounds. During fieldwork conducted by the researchers, it became evident that many tour guides in local training centers lack sufficient English proficiency to convey accurate information, which may compromise service quality and, consequently, tourist satisfaction. This underscores a context-specific gap in English language instruction tailored to the tourism industry, which remains underexplored in both local and regional contexts.

Despite the growing importance of English in global tourism, instruction in many tourism training centers remains inadequate. Courses often rely on lectures and rote learning, offering limited opportunities for learners to use English in authentic tourism-related situations. This study addresses a methodological gap by focusing on faculty perceptions rather than conventional learner-centered outcomes, thereby examining instructional practices through a qualitative lens that emphasizes experience, interpretation, and engagement with digital tools.

Although modern technologies have significantly advanced language learning, faculty members in tourism institutions continue to face challenges in effectively integrating these tools into their teaching practices. There is a notable gap in the meaningful incorporation of educational technologies, including e-learning platforms, artificial intelligence applications, and interactive digital tools. By investigating these challenges, the study provides conceptual originality, proposing a framework that links digital pedagogy with tourism-specific language instruction. This approach moves beyond generic studies on technology integration, concentrating instead on the unique intersection of digital tools, English language teaching, and tourism education.

These challenges highlight the need to explore how digital technologies are perceived and utilized in tourism education. Therefore, this study is framed as an exploration of faculty perceptions regarding the integration of digital technologies in English language instruction within tourism education. By shifting the focus from learner outcomes to faculty perspectives, the study captures the contextual and conceptual novelty of understanding how instructors interpret, experience, and engage with these tools in their pedagogical practices.

Specifically, the problem addressed in this study is the limited understanding of faculty perceptions concerning the integration of digital technologies in English language teaching for tourism education. This deliberate focus emphasizes the methodological and conceptual originality of the study. Accordingly, the research question guiding this inquiry is: *How do faculty members in tourism education perceive the integration of digital technologies in English language instruction?*

## Method

### Research design

This study adopted a qualitative research design, which is widely recognized as appropriate for exploring complex educational phenomena and capturing participants’ lived experiences and perspectives Denzin & Lincoln, 2018^48,49^. This approach enabled the collection of rich, in-depth insights from faculty regarding their technology-enhanced pedagogical practices in tertiary-level English for Tourism instruction.

The qualitative design was deliberately selected for its capacity to capture nuanced perspectives and instructional practices that may not be adequately revealed through quantitative methods, particularly in contexts where pedagogical transformation involves both conceptual understanding and practical application. As highlighted in qualitative research literature, this design is especially suitable for investigating meaning-making processes within educational settings^50^, Marshall & Rossman (2016).

A descriptive analytical framework was employed to examine narrative accounts and identify emerging thematic patterns in participants’ responses. This approach aligns with established qualitative analysis procedures that emphasize systematic interpretation of textual data to generate meaningful and trustworthy insights^51^. Through this methodological framework, the study provides a grounded understanding of how digital technologies shape the teaching of English for Tourism.

### Population and sample

The population of the study consisted of 223 faculty members (N = 223) working in tourism training institutions across Jordan. From this population, a purposive sample of 65 participants (n = 65) was selected, representing approximately 29% of the total population. Sample adequacy was further examined using Cochran’s formula at a 95% confidence level and a 5% margin of error. The finite population correction indicated an adjusted required sample size of approximately 64–66 participants. Accordingly, the selected sample is statistically adequate and appropriate for the purposes of this exploratory study.

The study involved faculty members selected based on their relevance and experience in teaching English for tourism, particularly tour guiding. Participants were academically qualified, with 65% (n = 42) holding a master’s degree and 35% (n = 23) holding a doctoral degree. The gender distribution was relatively balanced, with 36 males (55%) and 29 females (45%).

Participants also demonstrated varied teaching experience, with 15 (23%) having 1–5 years, 28 (43%) having 6–10 years, and 22 (34%) having more than 10 years of experience. This diversity strengthened the study by providing a broad range of pedagogical and technology-enhanced teaching perspectives.

Given the exploratory nature of the study and its focus on obtaining in-depth insights into participants’ experiences, data collection was also guided by the principle of data saturation. Accordingly, data collection and analysis proceeded simultaneously until no new themes or significant insights emerged from participants’ responses, indicating that thematic saturation had been achieved. This further confirms the adequacy and appropriateness of the selected sample size.

## Research instrument

Data were collected using a semi-structured interview protocol designed in a tabular format to elicit detailed responses regarding the use of digital technologies in English for tourism instruction. This format ensured consistency across key thematic areas while allowing flexibility to explore emerging issues and participants’ reflections. The instrument was developed based on English for Specific Purposes (ESP) frameworks, principles of digital pedagogy, and an extensive review of relevant literature.

Throughout this study, the term *digital technologies* is used as an umbrella concept encompassing digital tools, digital platforms, and digital resources employed to support English language instruction in tourism education. As all participating faculty members were proficient users of English and routinely used English as a medium of instruction in their teaching contexts, the interview protocol was developed and administered exclusively in English. No translation into Arabic or any other language was required, and no translated version of the interview protocol was produced. The English version of the interview protocol used for data collection is provided in the Appendix.

Content validity was established through a panel of 14 experts in language education, tourism education, and instructional design. Based on their feedback, the interview protocol was refined, and only four open-ended questions were retained according to three key criteria: relevance to the study objectives, clarity and comprehensibility for participants, and the ability to elicit rich, in-depth qualitative data without redundancy. The final protocol included the following questions: (1) How do digital tools affect learners’ self-confidence in the English for tourism classroom? (2) How do digital simulations influence learners’ linguistic and tourism-specific competencies? (3) Which digital resources are most effective in enhancing English proficiency for guiding tasks? and (4) What motivates learners to engage with digital tools for learning English in tourism contexts (see the Appendix).

To ensure methodological rigor, a pilot study was conducted with five faculty members selected through purposive sampling based on their expertise in language education, tourism-related instruction, and familiarity with digital learning environments. The sample size of five was deemed sufficient for pilot purposes to assess clarity, feasibility, and consistency of the interview protocol rather than to produce generalizable findings, in line with qualitative research guidelines. The same interview protocol was administered twice to the same participants with a 13-day interval to enhance the stability and reliability of responses. Collected data were independently analyzed by two reviewers, and inter-rater reliability was assessed using Cohen’s kappa coefficient, yielding a value of 0.93, indicating excellent agreement. Minor refinements were made based on pilot study findings to improve the clarity and flow of the interview questions.

### Data collection procedures

Semi-structured interviews were conducted face-to-face in the participants’ workplaces, following a consistent, ethical protocol. All 65 educators were briefed on the objectives of the study, assured of confidentiality, and provided with consent forms to sign. Interview sessions ranged between 55 and 63 min and were audio recorded with participant permission.

To foster open and honest dialogue, the researchers established rapport and emphasized the anonymity of responses by assigning numeric identifiers instead of names. Clarifications and paraphrasing were used during interviews to ensure accurate understanding of participants’ views. A transcript validation step was also incorporated, wherein participants reviewed their respective interview transcripts one week after transcription to ensure accuracy and completeness.

### Data analysis

Thematic analysis was conducted using an inductive, data-driven approach informed by grounded theory principles, allowing patterns, concepts, and categories to emerge directly from the data without imposing predefined theoretical frameworks^51^. This approach was justified by the exploratory nature of the study, which aimed to capture faculty perceptions in depth while enabling the systematic generation and refinement of conceptual categories. Accordingly, data collection and analysis were conducted concurrently in an iterative cycle, ensuring that emerging insights continuously informed ongoing interpretation.

The analysis began with repeated reading of all transcripts to achieve deep immersion in the data and to identify recurring meanings, expressions, and preliminary conceptual patterns. This familiarization phase provided a foundation for subsequent analytical procedures and ensured sensitivity to participants’ narratives.

Following familiarization, an inductive open coding process was applied. Transcripts were segmented into meaningful units (words, phrases, or sentences), which were coded according to their conceptual relevance (e.g., learner engagement with digital tools). Through constant comparison, codes were continuously reviewed, refined, merged, or differentiated to ensure conceptual clarity and internal consistency across the dataset. This iterative movement between data and emerging interpretations facilitated the gradual development of higher-order categories.

The development of themes followed a systematic process of clustering related codes and continuously comparing them across the dataset. Themes were refined through iterative analysis to ensure conceptual distinctiveness, such that each theme represented a clearly defined analytical dimension. Although partial overlaps naturally emerged due to the interconnected nature of the phenomenon, these were retained as reflective of the complexity of teaching practices in tourism education while maintaining clear analytical boundaries.

This process resulted in six final themes aligned with the study objectives:

- Enhancing self-confidence in tourism language instruction
- Using digital reality simulations for linguistic and professional growth
- Leveraging digital resources for English proficiency in guiding contexts
- Motivating learners through digital engagement
- Promoting classroom interaction in digital learning environments
- Using continuous digital assessment for language development

Following theme development, a detailed descriptive narrative was constructed by integrating thematic interpretations with illustrative excerpts from the interviews. While frequency counts were not the primary analytic focus, they were selectively used to support thematic prominence and enhance interpretive depth.

In parallel, theoretical saturation was assessed continuously through the constant comparison method. Each interview was analyzed immediately after completion, and emerging codes were compared with previously collected data. Saturation was considered reached when no new codes or conceptual dimensions emerged in successive interviews, indicating that the categories had become stable and theoretically well developed.

Saturation was specifically identified at interview number (53), where no new codes or themes emerged and data showed clear repetition. To enhance the credibility and dependability of this determination, data collection was deliberately extended to include an additional 12 interviews (total n = 65). This confirmatory step ensured that saturation was not an artefact of sample size. The additional interviews reinforced the initial conclusion, as no new conceptual insights emerged, confirming that saturation had already been achieved at interview (53), with further data serving only a verification function.

To enhance methodological rigor, two researchers independently conducted the coding process. Coding was performed separately and then compared to ensure consistency in interpretation. Any discrepancies were resolved through discussion and consensus to ensure analytical accuracy and interpretive coherence. Inter-coder reliability, calculated using Cooper’s formula, yielded an agreement coefficient of 0.94, indicating a high level of consistency and strengthening the dependability and trustworthiness of the analysis.

### Ethical considerations

Ethical approval and the necessary permissions were obtained from the Ethics Committee of King Faisal University (protocol code: KFU-REC-2024 Mar-EA000695; approval date: 12 March 2024), and the study was conducted in accordance with the Declaration of Helsinki. Since the study involved more than one institution, ethical approvals and permissions were also obtained from all participating institutions, ensuring compliance with the regulatory and ethical requirements of each site. The researchers adhered to all standard ethical research protocols, obtaining informed consent from all participants after they were fully informed about the study objectives, the voluntary nature of their participation, and the confidentiality of their responses, with the assurance that they could withdraw at any time without consequences. All interview transcripts were anonymized using numeric codes to protect participants’ identities, audio recordings were made only after obtaining verbal consent, and participants were invited to review their transcripts to verify the accuracy of the recorded information.

## Findings

This study examined the transition from traditional instructional approaches to the integration of digital tools in English language teaching within tourism education. Drawing on qualitative data from semi-structured interviews with 65 faculty members across academic and vocational institutions in Jordan, the analysis identified six key themes reflecting current pedagogical and technological practices. The findings illustrate how digital technologies are incorporated into English language instruction and how they are understood in relation to learners’ communicative competence and professional skill development in tourism contexts. The identified themes include enhancing self-confidence in tourism language instruction; using digital reality simulations to support linguistic and professional development; leveraging digital resources for guiding-specific language use; fostering motivation through digital engagement; promoting interaction in digital learning environments; and employing continuous assessment to support language development.

### Enhancing self-confidence in tourism language instruction

A large majority of participants (62 out of 65; 95.38%) identified self-confidence as a central element of effective English communication in tourism settings. They emphasized that linguistic knowledge alone is insufficient unless learners are able to communicate confidently in authentic professional contexts. In their accounts, self-confidence develops progressively through sustained practice, interaction, and exposure to communicative tasks.

Interactive digital tools, including role-playing applications and virtual simulations, were described as providing structured opportunities for meaningful practice. These tools were viewed as useful for engaging learners in realistic tourism scenarios, which may help reduce communication anxiety and support fluency development when appropriately integrated into instruction.

Participants further noted that such technologies enable learners to simulate professional tasks such as conducting virtual tours and responding to tourist inquiries. These activities were considered particularly relevant for preparing learners for workplace communication in tourism environments, although their effectiveness depends on instructional design, pedagogical strategies, and implementation conditions.

In addition, digital tools were reported to support the development of core language skills, listening, speaking, reading, and writing, through exposure to context-specific and task-based language use. From this perspective, self-confidence emerges as an important outcome of practice-oriented instruction, where digital technologies function as facilitators of engagement rather than direct determinants of learning outcomes.

Overall, the findings suggest that sustained engagement with simulated and interactive learning environments may enhance learners’ ability to communicate effectively, respond appropriately in tourism-related situations, and perform professional tasks in English, as illustrated in the interview excerpts below.

In our training program, we focus on developing personal skills alongside language proficiency. By using simulation technologies for tourism situations through interactive applications, we have noticed that students are more prepared to interact in English in real situations, which helps them build self-confidence and an ability to deliver professional tourism information.

Proper preparation for a tour guide includes mastery of the language as well as the ability to interact with tourists confidently. Students prepare for real-life situations by conducting virtual tours, answering tourists’ inquiries, or role-playing inquiries, which helps them overcome the language barrier and increases their self-confidence.

At our college, we appreciate the dual importance of language self-confidence and proficiency for a tourism guide. Role-playing activities in digital environments allow students to face tourists and equip them with the skill to interact naturally, positively impacting their capacity to perform tours in English confidently.

### Using digital reality simulations for linguistic and professional growth

The analysis indicated that 61 participants (93.84%) highlighted reality simulations as an effective pedagogical approach for supporting both linguistic development and professional competencies, particularly for learners preparing to guide English-speaking tourists. In their accounts, such simulations are viewed as providing structured opportunities for meaningful English practice within authentic-like tourism contexts.

Participants described digital simulations as enabling exposure to realistic communicative situations that mirror workplace demands, including guiding tasks, responding to tourist inquiries, and delivering informational content. Within this instructional perspective, simulations are seen as helping learners bridge the gap between theoretical language knowledge and applied professional communication.

At the same time, the value of these tools was considered to depend on appropriate pedagogical design, classroom integration, and implementation conditions. Rather than functioning as standalone solutions, digital simulations were understood as supportive instructional tools whose effectiveness is shaped by how they are embedded within teaching practice.

Overall, the findings suggest that reality-based simulations are perceived as an important component of digitally supported ESP instruction in tourism education, contributing to both language practice and the development of professional communicative skills, as illustrated in the interview excerpts below.

Integration of virtual tourism into practical training enhances students’ oral fluency to a great extent… In one assignment, we asked students to virtually tour a site and give an English tour using the appropriate techniques for 'Umm al- Jimal.' We realized that students tended to use the language more freely and more confidently because they were more advanced.

Undoubtedly, assigning students oral work and role play activities that portray and simulate real-life situations in tourism aids in self-assessment of their language mastery. We used to ask them to video-record some of their interactions with tourists and then critique their accent and grammar, which each week meant they performed better and better.

In our college, we consider that the preparation of a tourism guide goes beyond mere linguistic skills but the ease of expression linguistically and socially in English. Implementing role-playing exercises for students with digital applications for greeting and explaining sites to tourists, we observed a remarkable decrease in students’ language anxiety and an increase in their willingness to participate.

Integrating information technology for the simulation of professional situations provides us with the opportunity to practice language as a skill, and not just theory…. When students assume the roles of guides and explain the tours to their classmates or an avatar in an app, they have a chance to become fluent English speakers.

Participants’ responses indicate that digital reality simulations are viewed as supportive tools for developing both professional competencies and language fluency in tourism contexts. Virtual tours and role-playing platforms were described as creating practice-oriented environments that encourage meaningful language use and the application of tourism-specific vocabulary. These simulations also provide structured opportunities for interaction, which are considered conducive to the development of communication skills relevant to the tourism sector when appropriately integrated into instructional practice.

In addition, simulation-based activities were associated with enhanced learner confidence and engagement. Participants noted that learners who regularly engage with such tools may appear more prepared for fieldwork interactions compared to those with limited practice opportunities. Such observations reflect instructional experiences rather than directly measured outcomes.

Overall, digital reality simulations are perceived as useful pedagogical tools for reducing language-related anxiety and supporting communicative preparedness in tourism education, particularly when embedded within well-structured teaching and learning environments.

### Leveraging digital resources for english proficiency in guiding contexts

The analysis revealed that 58 participants (89.23%) indicated that faculty members in tourism education institutions use a range of digital technologies and digital resources to support learners’ English proficiency in guiding contexts. These practices were described as grounded in pedagogical experience and shaped by instructional needs, learner profiles, and available institutional resources, rather than being uniformly applied across all contexts. The following interview excerpts illustrate these perspectives:

Digital resources like tourism podcasts and interactive videos have become a core part of our curriculum. We ask students to listen to real interactions between tour guides and foreign tourists, then simulate those dialogues in class. This exercise alone helped them use the language confidently in field situations.

In one course, we integrated audio resources like tourism podcasts and video dialogues between guides and tourists. Students were asked to role-play in class. This approach helped them use English with greater confidence in real situations that mirror their future work.

We used the Future Learn platform to provide specialized content combining language and real tourism scenarios, such as interacting with visitors at historical sites. This enabled students to gain a deeper understanding of both the linguistic and professional context.

We evaluated students’ writing skills through Google Forms, where they wrote real-world texts like booking confirmation emails or descriptions of tourist attractions. This digital feedback improved their professional competence in using the language in tourism tasks.

Through the Learn English for Tourism platform, students created visual presentations on local destinations, relying on texts and activities from the tourism guiding profession. This enhanced their speaking skills in front of an audience, using precise and appropriate terminology.

These reflections indicate that faculty members in tourism education institutions draw on experience-based insights when using digital technologies and digital resources to support language instruction. Rather than presenting these resources as universally essential, they are viewed as complementary components that enhance instructional practice when aligned with course objectives and learner needs. Faculty reported adopting targeted strategies such as educational platforms, interactive content, digital assessment, and self-directed learning activities to support both general language development and tourism-specific vocabulary. These approaches were associated with increased engagement with context-relevant language, particularly when integrated into scenario-based learning activities.

In addition, participants emphasized the communicative demands of the tourism sector, highlighting the importance of preparing learners to use English in realistic, workplace-relevant contexts. Reported practices, including the use of platforms such as *Learn English for Tourism* and synchronous sessions (e.g., via Zoom) with industry professionals, reflect efforts to connect classroom instruction with professional communication settings, with outcomes depending on contextual implementation and pedagogical design.

Participants also described their role as facilitators who aim to expose learners to authentic tourism discourse through digital means. Overall, the integration of technology was portrayed as intentional and goal-oriented, supporting alignment between language learning and professional preparation in tourism education.

### Motivating learners through digital engagement

Out of the 65 participants, 53 (81.53%) indicated that instructors demonstrate a willingness to incorporate digital tools into their teaching practices. Participants noted that the integration of digital tools is associated with enhanced learner engagement and may contribute to language development when appropriately implemented. In preparatory tourism courses, these tools are used both for practicing language skills and for introducing professional communication tasks, while remaining complementary to broader instructional approaches rather than functioning as standalone solutions.

Participants further highlighted that the use of digital tools may influence both teacher and student motivation. These perspectives are illustrated in the following excerpts, which reflect how digital technologies are incorporated into tourism guide training contexts:

Students have become more willing to participate in class activities after the integration of digital learning tools, where we noticed improvements in their language performance, reflecting an increase in their intrinsic motivation to develop their skills.

Learning through interactive digital applications made students more enthusiastic about improving their English language skills, and increased their desire to use these skills in real-life situations, such as interacting with tourists.

Through the use of digital learning platforms, we observed that students no longer only receive knowledge, but actively seek opportunities to apply what they have learned, reflecting their intrinsic motivation and enjoyment of the learning process.

Integrating technology into tourism guiding instruction helped create a more engaging learning environment, where students showed increased interest in improving their language performance due to the realistic nature of the activities and their direct connection to the job market.

These excerpts indicate that learner motivation to engage with digital tools is considered by participants as an important factor in supporting language learning within tourism contexts. Rather than suggesting a direct causal relationship, motivation is described as being influenced by opportunities for active engagement and meaningful language practice. In this regard, digital resources are viewed as potentially encouraging greater learner participation and responsibility in the learning process.

Participants further noted that the use of digital tools may be associated with positive shifts in learner attitudes, including increased engagement, independence, and initiative. For example, one participant observed that students increasingly seek opportunities to practice English both in classroom settings and during field experiences, reflecting greater awareness of the practical value of language use.

In addition, some participants linked higher levels of motivation with improved communicative performance in tourism-related tasks, particularly those involving interaction with tourists. Activities such as creating digital tourism content were described as supporting learners’ understanding of the functional relationship between language and professional practice.

### Promoting classroom interaction in digital learning environments

The analysis of interview data revealed that 55 participants (85%) mentioned the importance of interaction in digital learning environments for language development among prospective tourism guides. Interaction was consistently identified as a core component of language learning, with digital environments viewed as extending opportunities for communication when effectively integrated into instructional practice.

Participants highlighted collaborative activities through digital platforms, discussion forums, and interactive workshops as approaches that support communication in tourism-related contexts. These practices were described as enabling greater participation and exposure to varied communicative situations, particularly in task-based and scenario-oriented learning environments. Their effectiveness, however, was understood to depend on instructional design and facilitation strategies.

In the digital learning environment, we divided students into workgroups to address specific tourism scenarios, such as providing virtual tours or greeting tourists. We used interactive platforms that allowed them to exchange ideas in English, which helped them develop their language skills in a realistic context that mirrors professional settings.

Through group interaction in digital workshops, we noticed that students became more open to using English fluently. These activities enhanced their speaking and listening skills and made them feel they were part of an active and interconnected learning community.

Collaborative digital activities, such as forums and group discussions, provided a motivating environment for students to apply English in tourism-related situations, such as handling tourist complaints or explaining historical landmarks. This helped them build genuine confidence in their skills.

We created a flexible virtual environment that allowed students to communicate in English outside of official lecture hours through interactive digital tasks. This continuous interaction played a pivotal role in improving writing and speaking skills, especially in situations requiring specialized tourism language.

These insights indicate that digital classroom interaction is viewed by participants as an important component in supporting the development of students’ linguistic proficiency in tourism education, rather than as a sole determining factor. Participants emphasized the importance of designing interactive learning environments that situate English use within realistic tourism-related scenarios. Such environments were described as enabling meaningful practice and engagement when appropriately structured.

The findings further suggest that participation in group work through forums, workshops, and collaborative digital tasks may contribute to the development of both language use and teamwork skills relevant to the tourism guiding profession. Participants reported that learners engage in applied tasks such as discussing tourism programs and evaluating visitor experiences, which reflect key aspects of professional practice. These activities are understood to support the connection between language use and real-world contexts, with their effectiveness depending on task design and instructional implementation.

In addition, interview data indicated that student interaction extends beyond asynchronous platforms to include synchronous sessions conducted via Zoom or Microsoft Teams. These sessions were described as providing opportunities for real-time communication and guided practice, where students prepare and present reports on tourist destinations using relevant terminology. Such practices are perceived as helping bridge theoretical learning and practical application, particularly when learners assume simulated roles such as tour guides. One participant noted that virtual explanations of tourist sites may contribute to developing professional identity and confidence.

### Using continuous digital assessment for language development

The analysis of interview data revealed that nearly all participants (98.5%) acknowledged the role of continuous assessment supported by digital tools in enhancing language development within tourism-specific contexts. Continuous assessment was viewed as a mechanism for monitoring learner progress and informing instructional decisions when appropriately implemented.

Participants noted that formative assessment methods, such as online quizzes and interactive tasks, enable instructors to track learners’ development and provide timely, targeted feedback. These approaches were described as contributing to more responsive and adaptive teaching practices, particularly in technology-enhanced learning environments. Their effectiveness, however, was understood to depend on alignment with learning objectives and integration within the overall instructional design. The following excerpts from the interview transcripts further illustrate these perspectives.

Digital assessment enabled me to track students’ progress in English language skills immediately and accurately, which allowed me to adjust training plans according to each student’s progress, whether in fluency, correct use of tourism-related terms, or oral interaction skills.

In one of the training programs I supervised, we used interactive assessment tools to measure communication skills, such as simulating real tourism scenarios through video clips and electronic pronunciation tests. The results helped us identify students who struggled with presentation skills, and additional training units were assigned to support them.

Thanks to digital assessment tools, we now have a clear understanding of individual differences among students. For example, one assessment revealed that a group of students needed additional support with the use of tenses in speaking, which led me to adjust group speaking activities to directly address this gap.

Electronic assessments provided us with the ability to give immediate feedback to students after every activity, whether it was a written test or a speaking task. This quick interaction with performance results boosted students’ motivation and opened up a continuous avenue for individual discussions about areas of improvement, especially in contexts requiring linguistic precision, such as providing explanations of tourist attractions.

Participant perspectives indicate that digital continuous assessment is a supportive pedagogical strategy for enhancing linguistic competence in tourism classrooms, rather than a standalone or determinative factor. The immediacy of digital feedback was described as enabling learners to reflect on their strengths and areas for improvement, thereby supporting ongoing engagement and self-regulation. Instructors also noted that such feedback can inform instructional adjustments, helping align teaching practices with learners’ needs and context-relevant language use.

The analysis further suggests that digital assessment facilitates differentiated instruction by helping educators identify specific learner difficulties and tailor support accordingly. Within this framework, the instructor’s role extends beyond evaluation to include guidance and facilitation, contributing to a more responsive and adaptive learning process aimed at preparing learners for professional communication in tourism contexts.

Overall, continuous technology-enhanced assessment is viewed as an important component of English language instruction in tourism education. Through digital feedback tools, structured tasks, and ongoing performance monitoring, learners develop language skills alongside increased awareness of communicative demands in tourism settings.

## Discussion

The interpretations presented in this section are grounded in participants’ instructional experiences and professional judgments rather than in independently measured learning outcomes. Accordingly, the discussion does not claim causal relationships between digital technology use and language development, but instead examines how faculty conceptualize and evaluate these pedagogical practices within their instructional contexts.

The findings indicate that faculty members in tourism education demonstrate strong awareness of evolving approaches to English language teaching, reflecting a shift from rote learning toward more purpose-driven and contextually grounded instruction (e.g.,^52,53^). Within this shift, English is increasingly positioned as a professional communication tool rather than a decontextualized subject. The study contributes to the literature by situating these developments within ESP and technology-enhanced language learning from a context-sensitive perspective.

A key finding is the integration of human-centered and technology-supported pedagogical practices aimed at developing communicative competence. This aligns with Communicative Language Teaching principles, which emphasize meaningful communication over isolated linguistic forms^7,54,55^. Digital tools were consistently described as facilitating simulation, practice, and contextualized language use, reinforcing prior evidence on technology-mediated language learning^56^.

Learner self-confidence emerged as an important factor influencing participation in communicative tasks. This aligns with research linking affective variables to willingness to communicate and to engage in language learning^57,58^. In tourism contexts, confidence was associated with greater readiness to interact, take communicative risks, and complete professional tasks, highlighting the role of affective dimensions in workplace-oriented language learning.

Simulation and role-play were identified as central instructional strategies for developing both linguistic and professional competencies. These approaches were considered particularly relevant for preparing learners for tourism-related communication demands, including interaction with international clients and real-world service scenarios. This aligns with ESP, Task-Based Learning, and CLIL frameworks, which emphasize authentic, goal-oriented language use^59–61^. In this study, these frameworks are used as interpretive lenses rather than as experimentally tested models.

Digital simulation tools, including virtual tours and role-play platforms, were viewed as effective in approximating workplace environments. Such tools were associated with experiential and active learning, enabling learners to engage in tourism-related communicative tasks such as guiding, responding to inquiries, and presenting information. This reflects constructivist principles of learning through meaningful engagement^62,63^.

Participants further emphasized the increasingly active role of learners in the learning process. This shift was described in terms of improved spontaneity, interpersonal communication, and professional readiness in tourism contexts, reflecting a move toward more practice-oriented and context-based instruction.

Digital technologies, including digital resources such as videos and podcasts and digital platforms used for interaction and content delivery, were considered integral to instruction rather than supplementary materials. Their use was associated with richer exposure to authentic language input and more diversified learning experiences, consistent with discussions on digital literacy in ESP contexts^64,65^.

Motivation and learner autonomy were recurrent themes, with increased engagement and initiative reported when digital tools were incorporated. These dynamics are conceptually linked to Self-Determination Theory, which emphasizes autonomy, competence, and relatedness as drivers of engagement^65,66^. Similarly, collaborative technologies such as discussion forums and video conferencing were viewed as supporting interaction, intercultural communication, and critical thinking in multilingual environments.

Digital assessment practices were described as supporting continuous formative feedback, individualized learning, and skill development. This reflects a broader shift toward learner-centered assessment approaches in tourism education^28,29^.

Overall, the findings suggest a gradual transition toward an integrated pedagogical model in tourism education, where language instruction, digital tools, and professional training are increasingly interconnected. English is positioned as a functional medium for workplace communication, and technology is viewed as a means of supporting applied, context-based learning.

When situated within the ESP literature, these insights align with prior studies emphasizing task-based and profession-oriented instruction in tourism education^1,67^. They also correspond with research highlighting the pedagogical value of digital tools in fostering contextualized and practice-oriented learning environments^56,60^.

However, a more meaningful comparison with the existing literature highlights an important distinction. Whereas many previous studies have reported measurable improvements in learners’ language performance following technology integration, the present study focuses on faculty perspectives on how these pedagogical practices are implemented and understood in tourism education.

Within this broader research context, the study contributes contextual originality by examining how ESP instructors in tourism education conceptualize and implement digital technologies within their educational and cultural settings. Rather than focusing on measurable outcomes, the study highlights how technology integration, workplace simulation, and communicative competence are understood and enacted within tourism-focused ESP instruction.

These insights align with existing literature advocating ESP curricula that emphasize applied communication, cultural literacy, and adaptive learning. In this framework, instructors are seen as facilitators who integrate technology with professional language use to prepare learners for tourism-related communication demands. Importantly, these interpretations are situated within the interpretive strand of ESP research rather than its experimental or evaluative strands.

From a theoretical perspective, the findings extend current discussions of ESP and technology-enhanced language learning by treating instructor perception as a mediating construct, through which digital pedagogy is interpreted, enacted, and adapted in professional contexts. This framing offers an original conceptual lens for understanding technology integration beyond traditional outcome-focused models.

Despite these contributions, the study has several limitations. First, the sample was limited to tourism education institutions in Jordan, which may restrict the contextual transferability of the findings. Second, the study relied exclusively on instructors’ perspectives, without incorporating learners’ views, classroom observations, or performance-based measures, limiting triangulation and contextual validation of how digital tools are enacted in practice.

From a practical standpoint, the integration of digital technologies in tourism education is influenced by contextual and institutional constraints, including limited infrastructure, unequal access to technological resources, and varying digital competence among instructors. These factors affect the feasibility, consistency, and sustainability of digitally enhanced pedagogical practices.

Methodological rigor was enhanced through systematic thematic analysis, inter-coder reliability procedures, and data saturation. Nevertheless, the qualitative nature of the study does not permit causal inferences or objective measurement of learning outcomes.

The findings provide a conceptual framework that can guide future pedagogical development and research design. Rather than offering direct evidence of effectiveness, the study identifies areas of pedagogical potential requiring further empirical investigation in authentic educational settings. Future research should adopt mixed-methods designs integrating instructor and learner perspectives, observational and performance-based data, along with longitudinal and experimental approaches to examine the impact of digital tools on language proficiency, communicative competence, and employability. Contextual variables such as institutional readiness, technological accessibility, and teachers’ digital competence should also be further explored to understand implementation conditions and constraints. Additional research is needed to examine the pedagogical potential of emerging technologies, including artificial intelligence, augmented reality, and mobile-assisted language learning, particularly within ESP and tourism education contexts.

Ultimately, the study underscores the need to reimagine English for tourism instruction through a digitally supported, professionally oriented, and learner-centered approach. While recommendations should be interpreted cautiously given contextual and implementation challenges, this approach has the potential to enhance graduate readiness by supporting both linguistic competence and cultural responsiveness in the evolving tourism industry.

## Conclusions and recommendations

The findings suggest that effective English language teaching in tourism education requires a shift from traditional, decontextualized instruction toward more integrated and digitally enhanced pedagogical approaches. Within this framework, digital tools were perceived as increasingly important in supporting learner engagement, cultural competence, and professional readiness. Accordingly, the findings address the research question through the lens of faculty perceptions rather than directly measured instructional outcomes.

In relation to this pedagogical shift, participants reported that digital technologies, including virtual simulations, mobile applications, digital assessment tools, and interactive learning platforms, may enhance learners’ linguistic development, including fluency, vocabulary use, pronunciation, and spontaneous interaction. However, these interpretations are grounded in instructors’ perceptions rather than independently collected student perspectives or performance-based data, which were not included in the study design. Consequently, the study cannot empirically verify the extent to which perceived improvements correspond to actual learner achievement.

Building on these interpretations, digital tools were further perceived as contributing to learner autonomy, confidence, and stronger connections between academic learning and professional application. Similarly, role-play activities, collaborative tasks, and guided reflection were viewed as potentially supporting both linguistic and interpersonal competencies required in tourism-related communication contexts.

Extending these findings, participants also perceived that digital platforms may facilitate self-directed learning by providing repeated, feedback-rich opportunities for communication practice in culturally diverse environments. Nevertheless, the effectiveness of digital integration appears to be context-dependent and influenced by several implementation-related factors, including limited digital infrastructure, insufficient institutional support, low levels of digital literacy among instructors and learners, and large class sizes. Under such conditions, digital integration may become less effective and may require substantial pedagogical adaptation to achieve meaningful learning outcomes.

Within this context, instructors were perceived as central to the successful integration of digital technologies, requiring both pedagogical expertise and digital competence. These findings highlight the importance of equipping educators with the necessary technological and instructional skills to implement digitally enhanced ESP instruction effectively. At the same time, these conclusions reflect perceived instructional practices rather than systematically observed teaching behaviors, reinforcing the interpretive nature of the study.

Moreover, the findings identify several barriers that may hinder effective digital integration in tourism education. These barriers include technological constraints, such as unstable internet connectivity and limited access to digital resources; institutional limitations, including insufficient training opportunities and limited administrative support; and pedagogical challenges, such as resistance to change and varying levels of digital literacy among instructors and learners. In such contexts, digital tools may function as supplementary resources rather than transformative pedagogical instruments, thereby limiting their educational impact.

In light of these findings, the study offers important implications for curriculum design, teacher professional development, and educational policy. At the curriculum level, ESP programs in tourism education should adopt more experiential, task-based, and digitally mediated approaches that reflect authentic communicative demands within tourism settings. Such curricula should integrate professional communication tasks, intercultural interaction, and technology-supported learning activities to better prepare learners for workplace realities.

Furthermore, at the teacher training level, continuous professional development programs are essential for strengthening instructors’ pedagogical and technological competencies, particularly in relation to the effective integration of digital tools into ESP instruction. These programs should focus not only on technical proficiency but also on pedagogical strategies for managing digitally supported, learner-centered environments.

At the policy level, educational institutions and decision-makers should prioritize investment in digital infrastructure and ensure alignment between tourism industry requirements and English language curricula. In addition, educational policies should support the development of learners’ communicative, intercultural, and digital competencies to meet the evolving demands of global tourism contexts.

### Limitations and future research

Despite its contributions, the present study has several limitations that should be considered when interpreting the findings. First, the sample was limited to tourism education institutions in Jordan, which may restrict the transferability of the findings to other educational, institutional, or cultural contexts. Consequently, the conclusions should be understood within the contextual boundaries of the study setting.

Building on this contextual limitation, an additional methodological concern relates to the nature and scope of the data sources employed. The study relied exclusively on instructors’ perspectives without incorporating learners’ views, classroom observations, or performance-based measures. As a result, the depth of triangulation and contextual validation remains limited, particularly in relation to how digital tools are enacted and experienced in actual classroom practice.

Within this interpretive framework, participants reported that digital technologies, including virtual simulations, mobile applications, digital assessment tools, and interactive learning platforms, may support learners’ linguistic development, including fluency, vocabulary use, pronunciation, and spontaneous interaction. However, these interpretations are grounded in instructors’ perceptions rather than independently collected student perspectives or empirically measured performance outcomes. Consequently, the study cannot verify the extent to which these perceived improvements correspond to actual learner achievement.

In this regard, the absence of student data and additional triangulating sources, such as classroom observations and performance-based assessment, represents an important methodological limitation related to data scope and triangulation. This limitation should therefore be considered when interpreting the findings, as the analysis reflects instructional perceptions rather than empirically verified classroom outcomes.

Extending from these methodological considerations, it is also important to acknowledge the practical and contextual constraints that may influence the implementation of digital technologies in tourism education. These constraints include limited infrastructure, unequal access to technological resources, unstable internet connectivity, and varying levels of digital competence among instructors and learners. Such factors may affect the feasibility, consistency, and sustainability of implementing digitally enhanced pedagogical practices across different educational settings.

Moreover, the effectiveness of digital integration may vary according to instructional and institutional conditions. In contexts characterized by large class sizes, limited technological support, insufficient professional training, or low levels of digital literacy, digital tools may function as supplementary rather than transformative instructional resources. Under such circumstances, meaningful technology integration may require substantial pedagogical adaptation and institutional support.

In relation to these implementation challenges, methodological rigor was nevertheless maintained through systematic thematic analysis, inter-coder reliability procedures, and data saturation. However, this form of validation remains procedural and interpretive rather than experimental in nature. While it supports the internal credibility and consistency of the findings, it does not establish causal relationships or objectively measured learning outcomes.

Given these limitations in scope, triangulation, implementation, and validation, the findings should therefore be interpreted as providing a conceptual and perception-based framework rather than empirical evidence of instructional effectiveness. Nevertheless, the study offers valuable insights into the pedagogical potential of digital technologies within tourism-focused ESP education and may inform future curriculum development and instructional design.

Building on this interpretive foundation, future research is recommended to adopt mixed-methods approaches that integrate both instructor and learner perspectives alongside classroom observations and performance-based measures. Longitudinal and experimental studies are also needed to examine the actual impact of digital technologies on learners’ language proficiency, communicative competence, and employability outcomes within tourism education contexts.

Furthermore, future investigations should examine contextual variables such as institutional readiness, technological accessibility, and instructors’ digital competence in order to better understand the conditions that facilitate or hinder successful implementation. Additional research is also needed to explore the pedagogical potential of emerging technologies, including artificial intelligence, augmented reality, and mobile-assisted language learning, particularly in relation to their applicability within ESP and tourism education environments.

Ultimately, the study underscores the need to reimagine English language instruction for tourism through digitally supported, professionally oriented, and learner-centered pedagogical approaches. However, such recommendations should be interpreted in light of the contextual constraints and implementation challenges identified in this study. Within these boundaries, digitally enhanced instruction nevertheless holds significant potential to support learners’ linguistic competence, intercultural communication, and professional readiness in the evolving global tourism industry.

## Data Availability

The authors will make the raw data supporting the conclusions of this article available upon request, without any undue restrictions. Requests for access to the raw data should be directed to Ali Ahmad Al-Barakat (aalbarakat@sharjah.ac.ae).

## Appendix

### Semi-structured interview schedule

**Title:** Digitizing ESP beyond chalk and talk in the English for tourism classroom.

**Type of Instrument:** Semi-Structured Interview Protocol.

### Introduction and participant briefing

**Dear Participant,**

Thank you for agreeing to participate in this study. This interview aims to explore faculty members’ perceptions and experiences regarding the integration of digital technologies in English for Tourism instruction, including their perceived influence on learners’ confidence, language development, professional competencies, engagement, and motivation.

Participation is voluntary. You may decline to answer any question or withdraw from the interview at any time without penalty. All information shared will remain confidential and will be used solely for research purposes. No identifying information will appear in any publication or presentation resulting from this study.

With your permission, the interview will be audio-recorded to ensure accurate transcription and analysis. All recordings and transcripts will be stored securely and accessed only by the research team. The interview is expected to last approximately 50–60 min.

There are no right or wrong answers; the study seeks to understand your professional experiences and perspectives regarding the use of digital technologies in English for Tourism education.

### Interview procedure

This semi-structured interview is guided by four core questions. The interviewer may use follow-up prompts when necessary to encourage elaboration and obtain richer descriptions of participants’ experiences. Participants are encouraged to provide examples from their teaching practice whenever possible.

### Final interview questions

**Q1: How do digital tools affect learners’ self-confidence in the English for Tourism classroom?**

**Follow-up prompts:**

- Can you describe situations where digital tools increased or decreased learner confidence?
- Do all learners respond similarly to digital tools? Why or why not?
- What classroom conditions influence this effect?

**Q2: How do digital simulations influence learners’ linguistic and tourism-specific competencies?**

**Follow-up prompts:**

- Can you provide examples of simulation-based or virtual learning activities?
- Which language skills are most developed through these tools?
- How do these simulations support tourism-related communication skills?
- What limitations have you observed?

**Q3: Which digital resources are most effective in enhancing English proficiency for guiding tasks?**

**Follow-up prompts:**

- Which tools, platforms, or applications do you find most effective?
- How are these resources used in guiding-related activities?
- What makes these tools particularly effective compared to others?
- Can you provide an example from your teaching experience?

**Q4: What motivates learners to engage with digital tools for learning English in tourism contexts?**

**Follow-up prompts:**

- What types of digital activities generate the highest motivation?
- How do learners typically respond to these tools?
- Are there differences in motivation among learners? Why?
- What factors increase or reduce engagement?

### Closing statement

Thank you for sharing your experiences and perspectives. Your contributions are greatly appreciated and will help advance understanding of the integration of digital technologies in English for Tourism education.

Before we conclude, is there anything else you would like to add that has not been covered in the interview?

If you have any questions about the study, please feel free to contact the research team.

## Declarations

### AI statement

This manuscript was prepared by the authors. AI tools (QuillBot and Grammarly) were used in a limited, supervised capacity for language polishing and proofreading only. These tools did not generate ideas, conduct analysis, or shape interpretation. The authors reviewed and verified all content and assume full responsibility for the accuracy, rigor, and conclusions of the research.
